# Sperm Quality Assessment in Honey Bee Drones

**DOI:** 10.3390/biology9070174

**Published:** 2020-07-18

**Authors:** Jesús L. Yániz, Miguel A. Silvestre, Pilar Santolaria

**Affiliations:** 1 BIOFITER Research Group, Higher Polytechnic School of Huesca, Institute of Environmental Sciences of Aragón (IUCA), University of Zaragoza, 22004 Zaragoza, Spain; psantola@unizar.es; 2 Department of Cellular Biology, Functional Biology and Physical Anthropology, University of Valencia, 46100 Burjassot, Spain; miguel.silvestre@uv.es

**Keywords:** *Apis mellifera*, male, reproduction, semen, sperm quality

## Abstract

The quality of honey bee drone semen is relevant in different contexts, ranging from colony productivity to pathology, toxicology and biodiversity preservation. Despite its importance, considerably less knowledge is available on this subject for the honey bee when compared to other domestic animal species. A proper assessment of sperm quality requires a multiple testing approach which discriminates between the different aspects of sperm integrity and functionality. Most studies on drone semen quality have only assessed a few parameters, such as sperm volume, sperm concentration and/or sperm plasma membrane integrity. Although more recent studies have focused on a broader variety of aspects of semen quality, some techniques currently used in vertebrates, such as computer-assisted sperm analysis (CASA) or multiparametric sperm quality testing, still remain to be developed in the honey bee. This may be attributed to the particular sperm morphology and physiology in this species, requiring the development of technologies specifically adapted to it. This article reviews the present knowledge of sperm quality in honey bee drones, highlighting its peculiarities and proposing future lines of research.

## 1. Introduction

Honey bees are one of the most important pollinators, playing a vital role in plant pollination of crop and wild species. For instance, it has been estimated that approximately 70% of all crop species worldwide are dependent on bees for pollination [1]. However, recent reports of high colony losses worldwide have raised public concern for the future of honeybees [2,3]. Regardless of the underlying causes of these losses, with many factors possibly involved [4], efficient reproduction is fundamental for replacing dead colonies.

A limiting factor of successful reproduction may be sperm quality. Since the queen will store viable sperm after insemination for several years, the study of semen quality in this species is especially relevant. It may determine the reproductive success of the queen and, as a consequence, the colony’s survival and level of productivity [5], as well as the success of artificial insemination (AI, also called instrumental insemination in this species) [6,7]. Poor semen quality generates poorer quality queens, this being considered one of the main causes of colony loss [8]. The study of drone sperm quality is also of considerable research interest. To date, it has been applied to study the effects of age [9,10,11,12], body size [13], genetics [10,12], temperature [11,14], nutrition [11], management [15,16], seasonal variations [10,17], disease [18,19], insecticides [20,21], miticides [22], semen storage in liquid and frozen states [9,23,24,25,26], semen handling [9,27,28], sperm competition [29] and physiology [30].

Despite its importance, considerably less research has been undertaken on honey bee drone semen quality than in the case of domesticated animals. For example, the numbers of articles on the “Web of Science” database from 1900 to May 2020 containing the keyword “sperm” was 4717, 1821, 982 and 252 in the bull, horse, goat and honey bee, respectively. Most studies on drone semen quality have only assessed a few parameters such as sperm volume, sperm concentration and/or sperm plasma membrane integrity. However, a proper assessment of sperm quality requires a multiple testing approach in order to discriminate between the different aspects of sperm integrity and functionality [20,25]. This article reviews the current knowledge of sperm quality in honey bee drones, highlighting its peculiarities and proposing future lines of research.

## 2. Normal Sperm Structure in the Honey Bee

**Figure 1:**
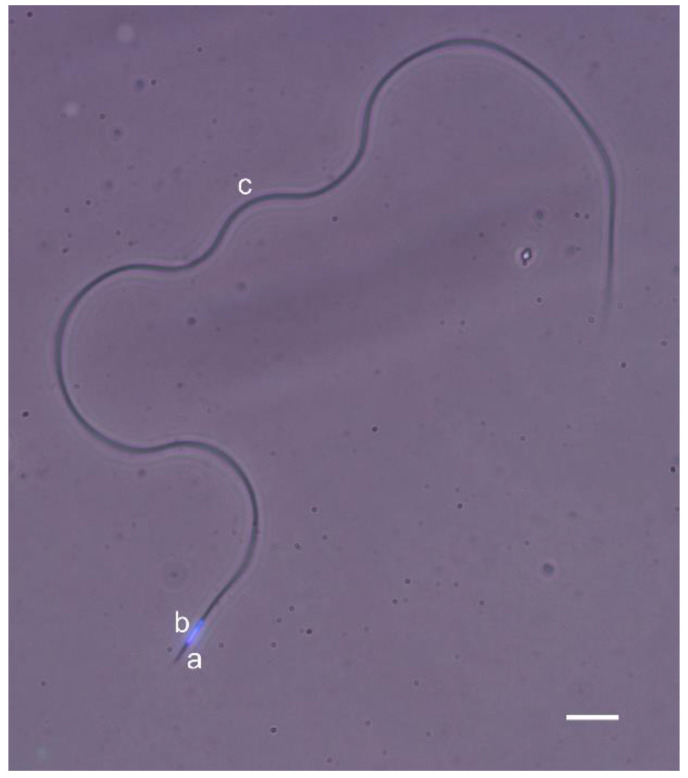
A combined phase-contrast and fluorescence (Hoechst) image of a honey bee spermatozoon showing the acrosome (a), nucleus (b) and flagellum (c). Scale-bar = 10 µm.

Honey bee sperm are long and filamentous cells with tapered ends (Figure 1) [31,32,33]. With a length of 250–270 µm and a width of 0.7 µm, the sperm consists of a relatively small and narrow head region (10 µm long and 0.4–0.5 µm in width [33]), the transitional centriole adjunct and the flagellum. The sperm head contains two consecutive parts of equal size: the acrosomal complex followed by a linear nucleus. The acrosomal complex is formed by a conical and two-layered acrosomal vesicle that covers the perforatorium up to the anterior nuclear end, where the perforatorium is inserted into a deep fossa [31]. The nucleus is dense and elongated, with a compact chromatin. The sperm flagellum is formed by an axoneme of 9+9+2 microtubular pattern, two large mitochondrial derivatives and two accessory bodies. The mitochondrial derivatives are asymmetrical in length and diameter and lie parallel to the axoneme throughout the sperm flagellum. The two accessory bodies are elongated structures located between the axoneme and each mitochondrial derivative.

## 3. Sperm Life Cycle in the Honey Bee

### 3.1. Spermatogenesis and Sperm Storage in the Male

Spermatozoa are produced in the drone testes from the larval to the pupal stages of development [34,35]. Between days 3 and 8 after emergence, the spermatozoa are transported from the testes to the seminal vesicles [34,36,37], where they are stored until ejaculation [37]. Immediately after emergence from their cells, drones are unable to copulate for about 9–12 days. However, there may be great variation in the time drones take to reach sexual maturity, which can depend on their genetics and the quality of the colony in which they are reared [10,16].

### 3.2. Mating, Sperm Storage in the Spermatheca and Egg Fertilization

Mating is the most significant function of an adult drone, although most of them fail in this task. Drones mate with the queen during the mating flight at the age of 15–23 days, 21 days on average [38]. In the afternoon of days with good weather [39,40], drones fly from the colonies to the male congregation areas, with a diameter of around 30–200 m, where thousands of drones from hundreds of colonies may await the arrival of a few virgin queens [41]. When a queen approaches a male congregation area, the drones chase her trying to copulate, forming a comet-like swarm in her wake. Normally, the queen mates consecutively with several drones in rapid sequence during the mating (nuptial) flight [10]. Earliest studies suggested that the queens copulate with 12–14 drones on average, usually in one or two mating flights [42,43,44,45], but a recent study suggested that the degree of polyandry might be much higher, so that queens can mate up to 34–77 males [46]. During mating, 6–12 million spermatozoa are transferred from the seminal vesicles of the drone into the genital orifice of the queen through the drone’s irreversibly everted endophallus [10]. After ejaculation, the endophallus is broken off inside the queen, acting as a temporary vaginal plug that may prevent sperm leakage, and the drone dies shortly afterwards. Approximately 10% of each male’s ejaculate is transferred to the queen’s oviducts [47] where the semen of the different drones is mixed [48].

After the nuptial flight, the queen returns to the hive, where the process of sperm transport from the oviducts to the spermatheca can take up to 40 h. Finally, only 3–5% of the received spermatozoa, 2 to 7 million, are usually stored [43,49,50], with an average of around 4–5 million sperm [51,52]. The queens do not mate again after the onset of oviposition [42], so that this sperm quantity must be enough to fertilize million eggs over their entire life span [53]. In the latter study it was also demonstrated that only two spermatozoa are required on average for egg fertilization.

## 4. In Vitro Evaluation of Semen Quality in the Honey Bee

### 4.1. Semen Collection

Two main techniques for collecting semen in drones have been described, based on the dissection of the seminal vesicles and the induction of ejaculation. In the earliest studies, semen was obtained mainly from the seminal vesicles (Table 1). In the last decade, however, induced ejaculation has been the main method used to collect semen samples.

Ejaculation of the honey bee drone is normally induced by the application of pressure on the thorax and abdomen. Semen is collected directly from the tip of the everted endophallus into a glass capillary tube connected to a syringe [76]. A higher success rate in semen collection may be expected in older rather than young mature drones (Table 2; [12]), and the results may be quite variable for different genetic lines and rearing conditions [10].

Unlike other domestic animal species, in which the evaluation of semen destined for insemination is a routine practice, in bees this assessment has usually been limited to research and toxicological studies, with little practical application associated to AI.

### 4.2. Semen Volume

Semen volume may be determined with the help of a precision syringe or by measuring the filled length of the capillary tube. Each drone will yield approximately 1 μL of semen, ranging between 0.1 and 2.4 μL [10,12,13,14,16,18,67,70,71,77]. It should be noted that the drones usually yield more semen than can actually be collected in the capillary tube [78]. Among the factors possibly influencing the volume of semen collected per drone, effects of age, body weight, season and breeding line have been described [10,77].

### 4.3. Sperm Concentration

Sperm concentration is one of the most frequently studied parameters of sperm quality in drones, allowing the spermatogenesis process and the sexual maturity of the drones to be evaluated [10]. Using AI, a direct relationship between the number of spermatozoa collected per drone and the percentage of them reaching the spermatheca has been described, so that individual differences in sperm quantity may be related to the drone’s paternity success [79].

The manual counting of sperm cells under a microscope with the help of a hemacytometer is by far the most frequently used method for sperm concentration assessment in the honey bee [12,18,20]. However, this approach is time consuming and provides variable results given the difficulty of obtaining a homogeneous distribution of spermatozoa in the sample and in the viewing chamber [20,80,81]. Special care should be taken in sample preparation using this technique. Spectrophotometers, commonly used to estimate sperm concentration in mammalian semen, have scarcely been studied for use with the honey bee [20,80]. It was demonstrated by Ciereszko et al. [20] that the spectrophotometer method may also be applied to this species, preferably using a 600 nm wavelength, to estimate the sperm concentration through the degree of light absorbance of the sample. However, the low volume of semen available in the honey bee requires the use of low-capacity cuvettes.

The use of other technologies described for determining sperm concentration in mammals, such as automated image analysis, flow cytometry, fluorescent plate reading or cell counters [82,83], have not been used in the honey bee. Given the particular morphology of the honey bee sperm, without a prominent head as in mammals, the identification of each spermatozoon by image analysis may be a difficult task. However, the combination of fluorescence microscopy and image analysis may allow rapid and accurate identification [84].

Sperm concentration of the drone semen usually ranges between 2 and 9 million sperm per µL. However, studies about this parameter in the honey bee usually refer to the total sperm quantity collected per drone, that is, the actual concentration multiplied by the semen volume. This parameter is highly variable, and differences of more than 100% have been reported between males [12,81].

In several studies, the spermatozoa were counted by dissecting the seminal vesicles of drones aged 11 or 12 days, with sperm numbers ranging from 1 to 30 million in individual drones (7.6 to 12.0 million sperm on average in the different studies (Table 2): [13,55,56,58,59,60,61]. The number of spermatozoa in the seminal vesicles may reach the highest level when the drones are around 7–9 days old [36,54]. The mean number of sperm collected per drone ejaculate is usually lower than that described for the seminal vesicles, ranging between 0 and 19 million (1.5 to 7.3 million on average in the different studies (Table 2): [10,12,64,70,71,77].

The sperm number produced per drone may vary according to body weight, age, season, genetics and disease [10,18,54,67,77]. Sperm production seems to be higher in autumn and from large and healthy drones around 21 days old. Large differences in the number of spermatozoa found in drones by different authors may also be explained by errors in the method used [81], inbreeding and the effect of different rearing and maintenance conditions of the drones [16].

The study of sperm concentration may also be applied to assess the reproductive quality of the queen, through the estimation of the number of sperm stored in the spermatheca [47,51,54,84]. This is an important parameter related to the longevity of the queen since, when the sperm reserve is depleted, queen bees are generally superseded (replaced by a young queen [85]) when they begin to lay unfertilized eggs that develop into drones [86]. The most frequently used criterion in assessing the reproductive quality of the young queen is to consider a threshold of 3 million sperm stored in the spermatheca to discriminate the quality of insemination [78]. The proportion of poorly inseminated queens (< 3 million sperm) ranged between 13.6% and 19.0% in the different studies [51,52,84,86,87]. Biotic, abiotic and management practices could affect the insemination quality of the queen [84]. The number of sperm in the spermatheca will logically be reduced with the queen’s age [52].

### 4.4. Sperm Motility

Although sperm motility is one of the most widely used parameters to determine sperm quality in mammals [88], in the honey bee it has been evaluated only occasionally [89]. However, sperm motility is a prerequisite for sperm migration to the queen’s spermatheca and for subsequent egg fertilization. In fact, it has been shown that sperm motility is more strongly correlated with sperm performance indicators in inseminated queens than other sperm quality tests, including the viability assay [25].

Most attempts to assess this parameter in the honey bee have been based on the establishment of a 4–6 grade score, according to the percentage of motile cells estimated subjectively [9,23,64,69,90,91,92,93]. In the more recent studies, sperm motility has been more frequently expressed as the percentage of motile cells, with some occasional indicators of the type of movement [20,25,74,89,94,95]. Spermatozoa may be classified as motile sperm if they present any type of active movement, freely motile if the sperm head shows displacement, and circular sperm if the sperm head and tail overlap [89]. Of these, total sperm motility seems to be a better indicator of semen quality than the other motility parameters, as it is less prone to bias due to uncontrolled variation in the experimental conditions [89]. Circular movement of sperm has been considered by some authors as an indicator of drone sperm quality [20,25]. We recently demonstrated, however, that this parameter shows a high sensitivity to the type of viewing chamber used and to the incubation period [89].

Until now, it has not been possible to develop computer-assisted sperm motility analysis in drones, due to difficulties in identifying sperm heads. Furthermore, honey bee sperm do not follow the typical motility patterns of mammalian sperm. Drone sperm tend to vibrate rapidly while moving in a relatively circular pattern [23]. It is therefore particularly important to reduce observer bias by repeated measurements.

A strict control over factors potentially affecting sperm motility is essential in order to obtain reliable results, and there is a need for standardization for each species [88]. The most commonly used method to assess sperm motility in drones is the use of slide coverslips, with or without sample incubation before or after loading it in the chamber. In a recent study, we demonstrated that the choice of viewing chamber and diluent used has a significant effect on the motility results and that traditional slide coverslips are contraindicated [89]. Specific disposable chambers seemed to give reliable results with negligible effects on sperm motility parameters, even when the measurement was made a long time after loading the chamber or using media without proteins. If the semen is diluted in media containing 2% BSA, the use of the Makler chamber may also provide reliable results. A minimum elapsed time of 5 min between chamber loading and sperm motility assessment was recommended for drones. The diluent used to assess honey bee drone sperm motility may also have a significant effect on the results [23,89].

Finally, it should be considered that it is unlikely that the in vitro study of the free swimming motility of drone spermatozoa reflects the in vivo situation, since certain flagellar beating patterns may be triggered by the mechanical restrictions inside the genital tract [96]. In this study, it was suggested that the double helical movement pattern of insect spermatozoa may be an adaptation to movement within narrow ducts, and that it is possible the axonemal wave would act against the duct wall rather than the surrounding fluid, leading to a crawling motion instead of swimming. This suggests that the use of specific microcapillary-based devices might be of interest in the study of sperm motility in this species.

### 4.5. Sperm Morphology

Unlike the case of mammals, for which the evaluation of sperm morphology is a fundamental task of semen analysis, very few studies have assessed the presence of morphologically abnormal spermatozoa in honey bee semen. Different aberrant tail forms, including coiled, frayed and double-ended forms, have occasionally been described in drones [97]. This may be attributed to a higher homogeneity of sperm forms in this species although, in the authors’ experience, the presence of abnormal forms may increase in certain circumstances, such as after freezing-thawing procedures. In fact, abnormal sperm heads have only been described in dead spermatozoa after freezing in liquid nitrogen [98]. Regarding sperm morphometry, total [77] and detailed [99] sperm length has also occasionally been included as a sperm quality trait, showing significant differences between the beginning and the end of the breeding season in the latter study.

### 4.6. Sperm Viability (Plasma Membrane Integrity)

Plasma membrane integrity is one of the most commonly assessed sperm quality parameters for semen analysis in the honey bee, as the loss of integrity of this thin outer boundary layer is considered incompatible with sperm viability [100]. Methods to assess the plasmalemma status are based on the increased permeability of damaged membranes to different substances, such as stains [57,69,93,101] or fluorescent probes [6,9,11,12,18,20,22,23,28,52,64,67,71,72,89,94,95,102,103,104]. The most commonly used fluorochrome for staining dead sperm cells is propidium iodide (PI). An intact plasma membrane is impermeable to PI, but this substance can pass through damaged membranes, bind to DNA and emit a bright red fluorescence in the nucleus of dead spermatozoa. It is often combined with a second dye, such as SYBR-14, Hoechst 33342 or acridine orange, that can cross intact plasma membranes of living sperm cells, and also have affinity to the DNA, emitting a green (SYBR-14, acridine orange) or blue (Hoechst 33342) fluorescence in living cells.

An alternative strategy is the use of hypo-osmotic solutions (hypo-osmotic swelling test, HOST), that may also provide valuable information on the plasma membrane status. The technique is based on the assessment of tail coiling associated with water accumulation in spermatozoa with intact plasma membranes [64,69,74]. Other methods based on cell enzymatic activity have also occasionally been used in the honey bee for viability assessment [15].

The manual counting of live and dead sperm cells under a fluorescence microscope after staining with SYBR-14/PI is the method most frequently described in the literature for sperm viability assessment in the honey bee (Table 2). Other technologies, such as flow cytometry [20,65,66,73,98] and image analysis [22,75], have also been implemented, allowing a more rapid evaluation of this parameter.

The results of sperm viability in fresh drone ejaculate is highly variable, ranging between 55% and 99% in the different studies (Table 2). In a step-by-step investigation, a reduction of about 10% of sperm viability in the sperm transport from the seminal vesicles of the drones (98.1%) to the lateral oviducts of the queen (88.7%) has been described [68]. This reduction naturally occurs during the second stage of eversion of the endophallus and during the injection of semen into the lateral oviducts of the queen, and has been attributed to increased pressure on the sperm during these stages. The sperm viability may also vary according to drone age, nutrition, management practices, environmental factors and exposure to insecticides and miticides [105].

The effect of mixing semen samples from multiple drones on sperm viability is controversial. In some studies, this procedure had no effect on sperm viability [29,66]. However, the dilution of semen in these studies may have reduced the possible deleterious effect of semen from different drones on sperm survival. In other studies, mixing semen samples had a negative impact on sperm survival [30,102]. Collins and Donoghue [102] observed that the semen processing procedure induced a greater physical stress on the spermatozoa in the pooled semen than on those of the individual ejaculates, which could explain the lower viability observed. In [30], semen was collected from the seminal vesicles and mixed with content from the accessory glands of other drones, showing a negative effect on sperm viability.

After storage in the queen’s spermatheca, sperm viability may also be highly variable, ranging between 20% and 100% in the different studies, with averages ranging between 80% and 98% [8,51,52,63]. A progressive loss of sperm viability with the increasing age of the queen has also been described [8,52].

### 4.7. Acrosome Integrity

During oviposition, the queen releases few spermatozoa from the spermatheca which enter the egg via the micropyles where they encounter the vitelline membrane. Then, the acrosome reaction releases lytic enzymes that aid in the penetration of the vitelline membrane to fertilize the egg. The integrity of the sperm acrosome is usually evaluated using fluorescently labelled lectins, plant proteins that specifically bind to some acrosomal glycoproteins. In the honey bee, the only study evaluating this aspect used *Pisum sativum* agglutinin (PSA) lectin staining, and was carried out on fixed and dead sperm, with full fluorescent acrosomes considered to be intact and acrosomes with lower or patchy fluorescent staining as damaged [74]. A disadvantage of PSA is that it shows less specificity to the acrosomal region than other lectins, such as *Arachis hypogaea* (peanut) agglutinin (PNA) [106,107]. Furthermore, PSA has affinity to egg yolk, which is commonly used in the diluents for drone sperm cryopreservation and non-specifically binds to the sperm surface. As a result, the acrosomal status may be evaluated incorrectly [106].

There is a need for more research about acrosomal integrity in the honey bee. The use of new fluorochromes and procedures should be evaluated. In mammals, the determination of acrosomal status in living sperm using flow cytometry or fluorescence microscopy is relatively common. The procedure is based on the fact that the lectins are large proteins that cannot penetrate an intact acrosomal membrane and, consequently, fluorescence is indicative of acrosome disruption or acrosome reaction and the absence of fluorescence is indicative of an intact acrosome [108,109,110].

### 4.8. Sperm Mitochondrial Function (Mitochondrial Membrane Potential)

Spermatozoa need energy to carry out their different functions and they can mostly obtain the ATP through the glycolytic and oxidative phosphorylation (OXPHOS) pathways [111,112]. There is increasing evidence that mitochondria play an essential role in regulating sperm function and lifespan, at least in mammals, for which the assessment of the sperm mitochondrial function is considered highly relevant [113]. It is assumed that mitochondria OXPHOS provide the primary energy substrates for the movement of sperm cells [114]. In insects, however, the role of the mitochondrial derivatives as energy-producing organelles has been called into question by several studies, although it has been assumed that they play an important biomechanical role in sperm motility [96].

Mitochondrial functionality is normally evaluated by its membrane potential. In the honey bee, [20] is the only study which has evaluated the sperm mitochondrial function using the probe Rhodamine 123 (R123) through flow cytometry. This was one of the first dyes used in mammals, which accumulates in the mitochondria emitting green fluorescence that varies in intensity depending on the number of functional mitochondria [114]. The disadvantages of this probe are its low sensitivity and its quick quenching time when compared to more recently developed probes such as 5,5,6,6-tetrachloro-1,1,3,3-tetraethylbenzimidazolylcarbocyanine iodide (JC-1) or the specific MitoTracker dyes. There is a need for further investigation into the sperm mitochondrial function in the honey bee, and the use of new dyes, such as JC-1 and MitoTracker, may help in this task [113].

### 4.9. DNA Fragmentation

Given the importance of the accurate transmission of genetic information to the offspring, several methods have been developed to detect damaged DNA in sperm [114]. These include the sperm chromatin structure assay (SCSA), the terminal transferase dUTP nick-end labelling (TUNEL) test, and the sperm chromatin dispersion (SCD) test. The TUNEL has been assayed in the honey bee to quantify DNA breakage caused by the cryopreservation procedure, although no clear increase was observed when compared to fresh semen samples [95]. Using the SCD test, a lower DNA fragmentation was observed in the sperm stored in the spermatheca than in the drone ejaculate [115]. The same technique was also used to demonstrate that *N. ceranae* infection causes sperm DNA damage in drones [116].

### 4.10. Sperm Apoptosis

Spermatozoa may exhibit certain characteristics of apoptotic somatic cells, such as DNA fragmentation, phosphatidylserine (PS) translocation, mitochondrial impairment or the presence of active caspases, as described in mammals [117]. Loss of plasma membrane asymmetry, especially translocation of phosphatidylserine (PS) from the inner to the outer leaflet has been studied in drone spermatozoa by annexin V staining, combined with the 7-Aminoactinomycin D (7-ADD) fluorochrome to detect dead cells [20]. However, the authors explained that apoptosis was not observed in the sperm samples using this fluorochrome combination.

### 4.11. Effect of Stress

#### 4.11.1. Oxidative Stress

The loss of redox homeostasis in sperm may generate oxidative stress that may have deleterious effects on the spermatozoa, including lipid peroxidation of the membranes and DNA damage [118]. In fact, spermatozoa are more susceptible to oxidative stress than somatic cells because they contain a reduced cytoplasm with few antioxidants and their plasma membrane is rich in unsaturated free acids highly susceptible to peroxidation. Furthermore, the high cellular metabolism of spermatozoa required to obtain flagella movement generates free radicals. This process may be aggravated by some treatments, such as dilution and cryopreservation [118].

The direct determination of oxidative stress through the analysis of reactive oxygen species (ROS) is very challenging due to their instability, the short lives of their intermediates [114] and the fact that spermatozoa intrinsically produce ROS [118]. The measurement of the defence capacity of sperm against oxidative damage and of the consequences of oxidative stress (lipoperoxidation of membranes and DNA damage) seem to be better alternatives. Some recent studies of the honey bee where the reduction potential of the cell [71] and of superoxide dismutase (SOD) activity [15] was analysed may be included in the first group. To our knowledge, there are no studies evaluating the lipid peroxidation of drone sperm membranes. This analysis may be done using boron-dipyrromethene (BODIPY) probes or specific antibodies against lipid peroxides [113].

#### 4.11.2. Response to Induced Stress

The response of spermatozoa to several kinds of induced stress, such as thermal [119], oxidative [120] or osmotic stress [121], has been used as a parameter of sperm quality in mammals. In the honey bee, the tolerance of drone sperm to osmotic and pH stress has also been evaluated, finding correlations with the number of spermatozoa reaching the spermatheca after AI [25].

### 4.12. Biochemical Assays

In addition to the aforementioned studies related to the antioxidant capacity, other biochemical assays, such as the quantification of adenosine triphosphate (ATP) content [15,71] and of enzyme-leakage from the cytosol and mitochondria [25], have also been occasionally used to determine the sperm quality in the honey bee. ATP is the main energy source used by the sperm for metabolic activity and motility [71]. Given its relationship with sperm motility, it may be initially argued that increased ATP concentrations may be indicative of better sperm quality [15]. However, an increase in energetic metabolism could lead to oxidative injuries by generating ROS, and the increased levels of ATP could be due not only to increased metabolism but also to decreased ATP consumption, resulting in lower sperm motility [71].

Biochemical tests to quantify the leakage of sperm enzymes after dilution and freezing-thawing may be used as markers for sperm cell damage. A significant negative correlation between the leakage of the glucose 6-phosphate isomerase (GPI) enzyme from damaged cells and the number of spermatozoa reaching the spermatheca after AI with cryopreserved semen has been described in the honey bee [25].

### 4.13. Multiparametric Sperm Quality Assessment

The measurement of multiple parameters simultaneously, cell by cell, may allow the development of more robust quality tests, and may expand research possibilities [113,122]. Various multiparametric tests may be developed nowadays, given the availability of a wide range of fluorescent probes, multichannel fluorescent microscopes and multi-laser cytometers. In fact, numerous combinations have been described in mammals [113], and similar methods are likely to be developed in the honey bee in the near future.

### 4.14. Contamination of Semen

Finally, contamination with microorganisms should be considered as an important aspect of semen quality, given its importance in terms of semen preservation and fertility, and the possibility of the transmission of diseases and infections to the queen. For instance, it has been demonstrated that semen samples contaminated with bacteria had a significantly lower mean viability than uncontaminated samples [9]. Semen may also contain Nosema spores and viruses that can potentially transmit diseases to the queen [123]. Given the relatively large size of Nosema spores (approximately 4.4 × 2.2 μm [124]), their presence may be checked microscopically when evaluating other parameters of sperm quality.

## 5. Conclusions

The study of sperm quality in the honey bee has many potential applications and research interest. However, several aspects of drone sperm physiology and quality still remain unclear and further investigation is needed about this topic. In this sense, the development of more sophisticated and objective methods, such as computer-assisted sperm analysis, the use of new fluorescent dyes and the development of multiparametric tests combined with flow cytometry and image analysis techniques would help in this task.

## Figures and Tables

**Table 1:** Mean values, sperm recovery techniques and staining used for the semen quality traits more frequently assessed in honey bee drones.

Origin of Semen *	Sperm Quantity (×10^6^/drone)	Sperm Viability (%) *	Reference
SV	9.9		[54]
SV	10.8		[36]
SV	8.5	-	[55]
SV	11.4	-	[56]
E		98.5 - E/N	[57]
E		86.9 - E/N	[9]
SV	8.6	-	[58]
E	8.7	99.2 - SYBR14/PI	[18]
SV	7.6	-	[59]
SV	9.2	-	[13]
E		78.1 - SYBR14/PI	[28]
SV	7.6		[60]
SV	12.0	-	[61]
E		81–88 - SYBR14/PI	[29]
SV	3.7–6.9		[62]
SV/E	7.3 (E)	98.1 (SV)- SYBR14/PI	[63]
E	3.2	-	[10]
E	1.5	87.2 - SYBR14/PI	[64]
SV		96.2 - SYBR14/PI	[65]
E		95.2 - SYBR14/PI	[66]
E		85.1 - Hoechst/PI	[25]
E	-	87.8–91.4 - SYBR14/PI	[67]
E		81.1 - SYBR14/PI	[22]
SV/E	-	98.1 (SV), 94.8 (E) - SYBR14/PI	[68]
E		88.4 - HOST	[69]
E	5.8	98.8 - SYBR14/PI	[16]
E	1.8	64.2 - SYBR14/PI	[12]
E	3.1	79.7 - SYBR14/PI	[70]
E	10.5	69.7 - SYBR14/PI	[71]
E		95–99 – 7-AAD	[20]
E		99.2 - SYBR14/PI	[72]
E		46.2–67.0 -SYBR14/PI	[73]
E		86.8 -HOST	[74]
E		70.6 - AO/PI	[75]

* SV: seminal vesicles; E: Ejaculate; E/N: Eosin/Nigrosin; PI: Propidium Iodide; HOST: Hypo-osmotic Swelling Test; AO: Acridine Orange; 7-AAD: 7-Aminoactinomycin D.

**Table 2:** Percentage of drones ejaculating semen after manual eversion.

Drone Age (days)	Ejaculation Success (%) Successsemen Collection Rate (%)	Reference
Mature	8.3–23.6	[73]
12	40.0	[18]
14	58.6	[10]
14	63.5	[12]
15	80.0	[16]
20	62.0	[71]
21	52.8	[10]
21–25	67.6	[70]
35	75.8	[10]
35	87.8	[12]
